# Philosophy of Science: How to Identify the Potential Research for the Day after Tomorrow?

**DOI:** 10.1100/tsw.2004.103

**Published:** 2004-06-25

**Authors:** Søren Ventegodt, Joav Merrick

**Affiliations:** ^1^ The Quality of Life Research Center, Teglgårdstræde 4-8, DK-1452 Copenhagen K, Denmark; ^2^ National Institute of Child Health and Human Development, Office of the Medical Director, Division for Mental Retardation, Ministry of Social Affairs, Jerusalem and Zusman Child Development Center, Division of Pediatrics and Community Health, Ben Gurion University, Beer-Sheva, Israel

**Keywords:** quality of life, QOL, philosophy, human development, public health, Denmark, Israel, philosophy of science, European Commission

## Abstract

We were asked to participate in a workshop to assist the European Commission on how to choose financial support for high-potential, basic research projects that can give new scientific breakthroughs and thus contribute significantly to the positive development of society, industry, and economy for the day after tomorrow. At this workshop, we analyzed the problem in some detail using experience from our own research on the global quality of life. We would suggest that the most promising projects have the following characteristics: (1) they are led by a brilliant researcher who considers his/her research to be “sweet science”, who wants to explain the anomalies of his/her field of science, and who lives in a nonmainstream scientific paradigm; (2) they are deeply engaged in the philosophical problems of their research field, they are searching eagerly for a new understanding and a new theory, giving new tools for measurement and creating change, and results are taken as feedback on all levels from tool to theory and philosophy; (3) they are focused on the key point(s), which is an essential feature of the universe that creates global change if intervened upon. At the NEST Pathfinder 2005 Topic Identification Workshop, Brussels 28 May 2004 entitled “Measuring the Impossible”, we advised the European Commission Research Directorate to allocate funds for projects focusing on the state of consciousness — how to understand it, how to map it, and how to develop it.

## INTRODUCTION

The European Commission Research Directorate has recently conducted a workshop on new and emerging science (Brussels May 28, 2004) in order to identify new activities in the scientific and technological sphere (New and Emerging Science and Technology or NEST) under the headline "Measuring the Impossible". The purpose was also to support unconventional and exploratory research of an interdisciplinary and/or multidisciplinary nature. We believe the subject of relevance to a broader public and have therefore decided to publish our reflections.

## RESEARCH FOR DAY AFTER TOMORROW IS MADE BY INDIVIDUAL PIONEERS

Research is made by researchers. This seems to be a tautological statement, but it is true about the more futuristic, controversial, or risky research, the more is it done by individual researchers instead of by a large group of researchers in the established scientific community. The pioneers are always few and the geniuses among them even fewer. The difficult question of supporting the research of the future is to find and support the individual researchers who are brilliant with projects that carry a strong potential for a major breakthrough in the future. These researchers have some common characteristics: they are out of pace with their time as their ideas and perspectives are for tomorrow or even for the day after tomorrow, they have a great passion for science, and they come from science for the sake of science itself. Francis Harry Compton Crick (1916–), the British scientist who received the Nobel Prize in 1962 in Medicine and Physiology, called it the "sweet science". These people live and breathe science. They are, in fact, science themselves.

These people are seldom appreciated fully by the scientific society, as their interest in new research paradigms[1] often makes them controversial and of little use in producing what is contemporarily seen as good and valid mainstream science. They are ahead of their time and the question often is if they really are crackpots or the prophets of tomorrow. If society, therefore, is interested in supporting basic research, the goal must be to find these young brilliant students or researchers in order to support them in their work.

## MEASURING THE IMPOSSIBLE TAKES A ROCK-SOLID STRATEGY

In the Danish Quality of Life Survey 1990–2004[2,3,4,5,6,7,8,9,10,11,12], we believed that a strong focus on the philosophy of science and research methodology[13,14,15,16,17,18,19,20] was necessary in order to measure the global quality of life[21]. We understood early in the project that in order to bring science a new step into the unknown, we had to express our understanding of this topic philosophically[22,23,24,25,26,27,28,29], understand the essence of the philosophy, and create formal theories[21,30,31]. By applying these theoretical tools and measurements in the clinical setting[32,33,34], we received feedback on many levels: on the measuring tools (the questionnaires[35,36]), on the developmental tools, on the theories, and on the philosophy (Fig. 1).

With a new philosophy[37,38,39,40], we could then formulate a new series of theories[41,42,43, 44,45,46,47], new tools for measuring[48] and creating the change[49,50], thus leading the team to a completely new research program[51]. This could give us completely unexpected results, such as how we are able to help HIV and cancer patients with new technology[52,53].

As Kuhn has pointed out[1], the scientific results will often only confirm the correctness of the philosophy and theory for a researcher who is busy producing papers and with meeting the needs of the world with his/her science. This busy schedule makes him/her ignore the most difficult, strange, and disturbing anomalies of the research (with an example from our own research: the spontaneous remission of metastasized cancer with a few patients, while most patients die[53]). The same results and anomalies will lead the pioneer into deep contemplation, a new understanding, and a theory (a new research paradigm) that is often highly unexpected (but valuable) results — the scientific breakthrough (i.e., learning what is necessary to induce the spontaneous remission[53]).

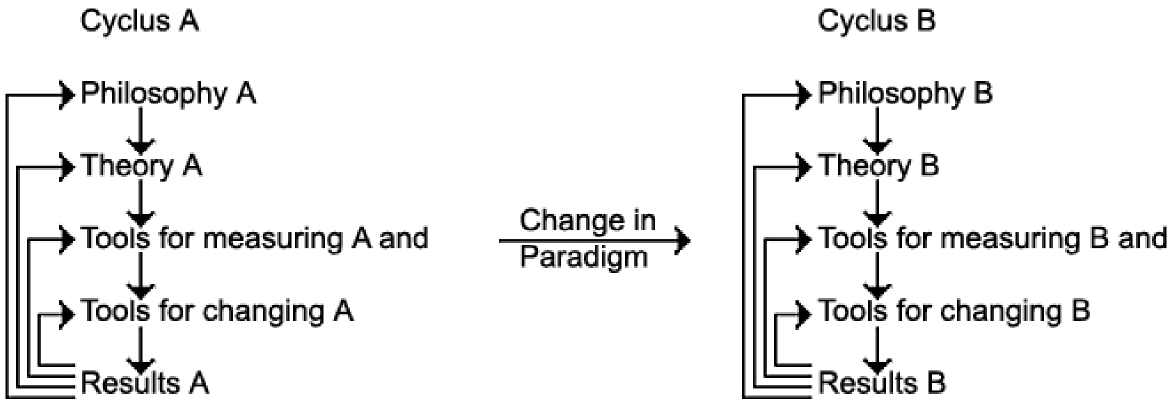

**FIGURE 1.** To measure the impossible — a new and unexplored area of reality (A) — takes understanding expressed as philosophy of A, new theory of A derived from the philosophy, new tools for measuring A, changing A derived from the theory, and results produced by the use of the combined tools, giving feedback to all levels above. While the results will confirm the correctness of the philosophy and theory for the normal researcher, ignoring the anomalies of the research, the same results and anomalies will lead the pioneer to new understanding and theory (a new research paradigm) and often to highly unexpected results — the scientific breakthrough.

So, what characterizes the project a high risk, high-potential research project? We believe that such a project always is designed with a strong focus on the fundamental philosophy of the research subject, with eyes wide open for the anomalies of the field of the research.

## FOCUSING THE RESEARCH

At the NEST workshop, we learned that the European Commission Research Directorate will support research “with urgency and potential for future societal, industrial, or economic relevance”. The problem is how to pick the research projects and the scientists most likely to contribute on this large scale. To optimize this probability, it is important to focus on the possibility for change and what creates it.

If we look at the three large areas of interest for the European Commission Research Directorate, we have visualized it in Fig. 2. This is only one way of many to focus on future research, but the way we would like to look at it with the knowledge we have gathered over the last decade with the fact that to us it seems that quality of life, health, and ability in general are primarily determined by consciousness[2].

## CONCLUSION

The research project(s) most promising and of importance for the day after tomorrow is the project(s) that meets the following criteria:

1. It must be led by a brilliant researcher who considers his/her research to be “sweet science” and who is deeply involved with the philosophical problems of his/her field.
2. The project must aim at developing new theories to explain the anomalies of the field and create new tools for measurement and intervention or, in other words, come from a new (nonmainstream) research paradigm.
3. It must be focused on a key point or an essential feature that can create global change if intervened upon. We suggest that the most promising key point is the state of consciousness — how to understand it, how to map it, and how to develop it[2].

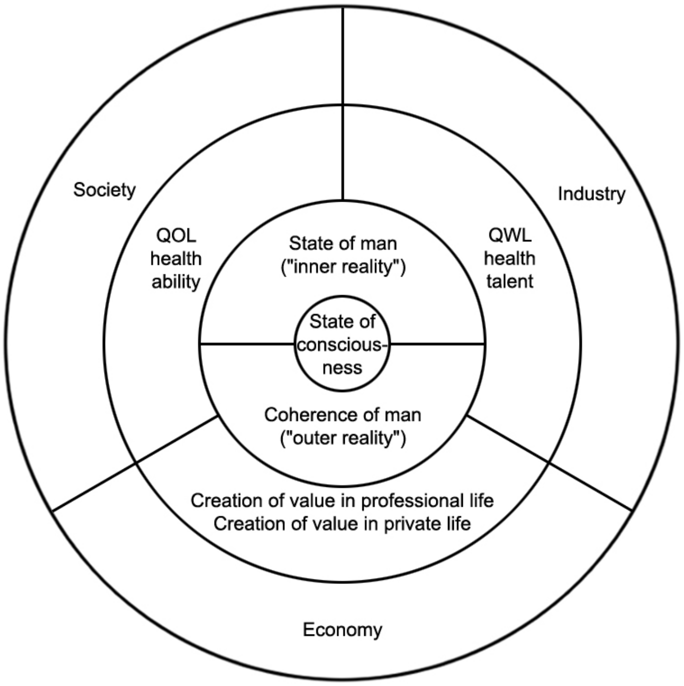

**FIGURE 2**. If you want to make basic science likely to give a major scientific breakthrough that will improve society, industry, and economy in the future, you are most likely to succeed if you find a key-point-project, a research project that focuses on how to understand and how to develop an important and causal aspect of the world; the state of consciousness seems to be such a key point[22,23,24,25,26,27,28,29,48].

We strongly believe that the most direct way to highlight unexpected scientific results and new scientific breakthroughs that will contribute to the positive development of society, industry, and economy is to support the researchers working with such projects *in person*. We also recommend that after the researcher is identified and the nature of his/her commitment is analyzed through peer review to secure that the criteria are met, he/she should have full control over the funding and be completely free to follow his/her own path of the research. One research cyclus (see Fig. 1) of the Danish Quality of Life Survey cost about 5 million EURO and with this knowledge in mind, we would recommend a grant size of 2–10 million EURO for a period of 3–10 years for comparable projects.

## ACKNOWLEDGMENT

This paper was supported by grants from IMK Almene Fond.

## BIOSKETCHES

**Søren Ventegodt, MD,** is the Director of the Quality of Life Research Center in Copenhagen, Denmark. He is also responsible for a Research Clinic for Holistic Medicine in Copenhagen and is a popular speaker throughout Scandinavia. He has published numerous scientific or popular articles and a number of books on holistic medicine, quality of life, and quality of working life. His most important scientific contributions are the comprehensive SEQOL questionnaire, the very short QoL5 questionnaire, the integrated QOL theory, the holistic process theory, the life mission theory, and the Danish Quality of Life Research Survey, 1991–94 in cooperation with the University Hospital of Copenhagen and the late pediatric professor Bengt Zachau-Christiansen. E-mail: ventegodt@livskvalitet.org. Website: http://www.livskvalitet.org

**Joav Merrick, MD, DMSc,** is Professor of Child Health and Human Development affiliated with the Zusman Child Development Center, Division of Pediatrics and Community Health at the Ben Gurion University, Beer-Sheva, Israel; the Medical Director of the Division for Mental Retardation, Ministry of Social Affairs, Jerusalem; and the Founder and Director of the National Institute of Child Health and Human Development. He has numerous publications in the field of child and human development, rehabilitation, intellectual disability, disability, health, welfare, abuse, advocacy, quality of life, and prevention. Dr. Merrick received the Peter Sabroe Child Award for outstanding work on behalf of Danish Children in 1985 and the International LEGO-Prize ("The Children's Nobel Prize") for an extraordinary contribution towards improvement in child welfare and well being in 1987. E-mail: jmerrick@internet-zahav.net. Website: www.nichd-israel.com
